# Social Influence on Risk Perception During Adolescence

**DOI:** 10.1177/0956797615569578

**Published:** 2015-05

**Authors:** Lisa J. Knoll, Lucía Magis-Weinberg, Maarten Speekenbrink, Sarah-Jayne Blakemore

**Affiliations:** 1 Institute of Cognitive Neuroscience; 2 Department of Experimental Psychology, University College London

**Keywords:** development, adolescence, social influence, peer influence, conformity, risk perception

## Abstract

Adolescence is a period of life in which peer relationships become increasingly important. Adolescents have a greater likelihood of taking risks when they are with peers rather than alone. In this study, we investigated the development of social influence on risk perception from late childhood through adulthood. Five hundred and sixty-three participants rated the riskiness of everyday situations and were then informed about the ratings of a social-influence group (teenagers or adults) before rating each situation again. All age groups showed a significant social-influence effect, changing their risk ratings in the direction of the provided ratings; this social-influence effect decreased with age. Most age groups adjusted their ratings more to conform to the ratings of the adult social-influence group than to the ratings of the teenager social-influence group. Only young adolescents were more strongly influenced by the teenager social-influence group than they were by the adult social-influence group, which suggests that to early adolescents, the opinions of other teenagers about risk matter more than the opinions of adults.

Risk assessment is part of people’s daily lives. Should they cross a street on a red light? Should they make a phone call while driving or not wear a seatbelt for a short drive? People are constantly making decisions about whether they should or should not take such risks. Adolescence is a developmental period that has been associated with heightened risk-taking behavior, such as alcohol and tobacco use, unsafe sexual behavior, and dangerous driving (Eaton et al., 2012; Steinberg, 2008). Risky decision making is linked to a situation’s perceived riskiness (Mills, Reyna, & Estrada, 2008; Spurrier & Blaszczynski, 2014); for decades, heightened risk taking in adolescence was explained by the notion that adolescents were more likely than adults to perceive situations as controllable or less likely to perceive situations as harmful (Elkind, 1967; Lapsley, Milstead, Quintana, Flannery, & Buss, 1986). However, recent studies dispute the notion that adolescents are unaware of potential risks and feel invulnerable. For example, compared with adults, adolescents sometimes overestimate rather than underestimate risk (Millstein & Halpern-Felsher, 2002), and they perceive certain situations as more risky and harmful than adults do (Cohn, Macfarlane, Yanez, & Imai, 1995). Thus, a reduced perception of risk may not be the critical factor in adolescent risk taking.

Social influence is a factor in adolescent risk taking: Adolescents are more likely to engage in risky behavior (including dangerous driving, substance abuse, and shoplifting) when they are with their peers than when they are alone (Dishion & Tipsord, 2011; Gardner & Steinberg, 2005; Simons-Morton, Lerner, & Singer, 2005). Adolescents spend more time with their peers than with their families (Brown, 1990) and are particularly sensitive to peer rejection (Peake, Dishion, Stormshak, Moore, & Pfeifer, 2013; Sebastian et al., 2011; Somerville, 2013). Social influence is the subject of a large body of research that has demonstrated how readily participants are affected by the opinions, judgments, and behavior of other people (Klucharev, Hytönen, Rijpkema, Smidts, & Fernández, 2009; Walker & Andrade, 1996; Zaki, Schirmer, & Mitchell, 2011). Despite extensive research on social influence in adults, developmental patterns of social influence, especially social conformity in adolescence, are not fully understood.

Deutsch and Gerard (1955) identified two types of conformity in adults. First, conformity can result from the pursuit of accuracy (informational influence); studies have shown that adults tend to follow other people as a guideline in novel or unfamiliar situations (Hilmert, Kulik, & Christenfeld, 2006; Jetten, Hornsey, & Adarves-Yorno, 2006). Second, conformity can arise from the pursuit of acceptance (normative influence); adults yield to social influence to avoid rejection (Deutsch & Gerard, 1955). Normative conformity might be prompted by an awareness of social norms; that is, people make decisions on the basis of expected or acceptable behavior in social interactions (Cialdini & Trost, 1998). We were interested in social influence on adolescent risk perception, and in the current study, we specifically investigated whether the degree of social conformity varied depending on the origin of the social influence.

We investigated the development of social influence on risk perception from late childhood through adulthood. Five hundred and sixty-three participants rated the riskiness of everyday situations and were then shown how other people, either teenagers or adults, perceived the riskiness of the same situation (these other ratings were actually generated randomly). The participants were then asked to rate the situation again. We investigated two hypotheses: (a) Risk perception is influenced by the risk ratings of others and this social-influence effect decreases with age (*social-influence hypothesis*; Engelmann, Moore, Capra, & Berns, 2012; Walker & Andrade, 1996); and (b) this socially driven change in risk perception depends on whether the social-influence group consists of teenagers or adults (*peer-influence hypothesis*). Regarding this second hypothesis, we predicted that adults would be more influenced by the ratings of other adults than by the ratings of teenagers and, in contrast, that adolescents would be more influenced by other teenagers than by adults (Somerville, 2013; Stallen, Smidts, & Sanfey, 2013). This prediction is based on the proposal that the pursuit of social acceptance by peers is a significant determinant of adolescent decision making (Blakemore & Mills, 2014; Reyna & Farley, 2006).

## Method

### Participants

Participants were visitors to the Science Museum in London on 14 days in April 2013. Data from 563 visitors (mean age = 23.4 years, *SD* = 12.2, age range = 8–59 years; 313 females, 250 males) were included in the analyses. Data from 63 additional participants were excluded because their responses were incomplete or they reported developmental conditions, including autism, attention-deficit/hyperactivity disorder, epilepsy, dyslexia, dyspraxia, dyscalculia, or depression. Participants were divided into five age groups as in similar prior studies (Van Leijenhorst et al., 2010): 106 children (mean age = 9.6 years, age range = 8–11 years; 54 girls, 52 boys), 68 young adolescents (mean age = 12.9 years, age range = 12–14 years; 39 girls, 29 boys), 52 midadolescents (mean age = 16.6 years, age range = 15–18 years; 30 girls, 22 boys), 136 young adults (mean age = 24.7 years, age range = 19–25 years; 77 women, 59 men), and 201 adults (mean age = 37.1 years, age range = 26–59 years; 113 women, 88 men). Twenty-nine percent of participants (2% of children, 11% of young adolescents, 30% of midadolescents, 40% of young adults, and 36% of adults) were nonnative speakers of English, but all spoke English fluently. Informed consent was obtained from parents for participants under 18 years old and from adults themselves. The study was approved by the University College London ethics committee.

### Design

We used a 3 × 5 factorial design with the within-subjects factor social-influence group (teenagers, adults, and control) and the between-subjects factor age group (children, young adolescents, midadolescents, young adults, and adults).

### Risk-perception task

**Fig. 1.**
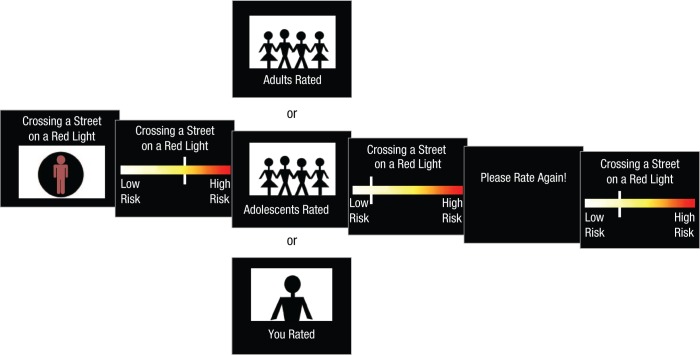
Illustration of the trial sequence. Participants were asked to imagine that someone was engaged in an activity (in this example, crossing the street on a red light). They then rated the activity’s risk by using a computer mouse to move a slider on a visual analogue scale. There was no time restriction for this first rating. After making this rating, participants were shown (for 2 s) a risk rating of the same situation that was ostensibly provided by a group of either adults or teenagers (the social-influence conditions) or was the participant’s own rating (control condition). The ratings from the social-influence groups were actually randomly generated. Finally, participants were asked to rate the same situation again. There was no time restriction for the second rating. The next trial started after 1 s.

In the risk-perception task, participants were presented with risky scenarios (see the Supplemental Material available online). The scenarios were generated on the basis of the following criteria: (a) They included a potential immediate health risk, such as an accident (e.g., crossing a street while texting, cycling without a helmet, driving without a seatbelt, climbing on a roof); (b) they elicited a relatively large amount of variance across individuals in terms of perceived risk; (c) they were generally considered to have moderate or medium risk; and (d) they included a variety of situations and avoided repetition. Stimuli consisted of single statements and were presented both aurally and visually. The auditory stimuli were spoken by a female English speaker and recorded in a soundproof chamber. After recording, stimuli were digitized (sampling rate = 44.1 kHz; bit depth = 16; monaural) and normalized. Statements were simultaneously presented aurally and displayed at the top of a screen, and they were illustrated with an image depicting the situation without providing too much contextual information (Fig. 1). The images were included to make the stimuli more appealing for museum visitors.

Participants read and listened to instructions before the main task, which started only after they pressed a button to indicate they had understood the instructions. The task was programmed using Cogent 2000 (University College London Laboratory of Neurobiology; http://www.vislab.ucl.ac.uk/cogent_2000.php) and run in MATLAB (Version R2012b; The MathWorks, Inc., Natick, MA). The entire task took around 12 min to complete.

Before the trials began, the participant’s age was displayed for 3 s alongside a message stating “Calculating your age group. . . .” The intention of this manipulation was that each participant would associate him- or herself with a social-influence group of a particular age (teenagers or adults). In addition, participants over 18 years old were explicitly told that the adult group picture represented a group of people their age, and participants between 12 and 18 years old were explicitly told that the teenager picture represented a group of people their age. Note that children’s ratings were not included for three reasons: First, our hypothesis pertained to adolescent risk perception and whether this was influenced differentially by risk ratings from other teenagers versus those from adults; second, we wanted to constrain the complexity of the experimental design; and third, we wanted to keep the study as short as possible because there were time limits for testing in the Science Museum.

On each trial, participants were asked to imagine that someone was engaged in the activity presented. The participants rated the activity’s risk by using a computer mouse to move a slider to the left side (low risk) or to the right side (high risk) of a colorful visual analogue scale. The slider initially appeared at a random position on the scale on each trial to avoid any consistent anchoring bias. There was no time restriction for the first rating. After making the first rating, participants were shown a risk rating of the same situation by either adults or teenagers (i.e., the social-influence group) for 2 s. These ratings were ostensibly from other participants; in fact, however, they either were randomly generated (adult social-influence condition, teenager social-influence condition) or were the participants’ own ratings (control condition). This minor deception was approved by the ethics committee. After this, the participant was asked to rate the same situation again. There was no time restriction for the second rating.

Participants provided the second rating in each trial, and then the next trial started after 1 s. A total of 79 different situations were generated; 18 (6 per social-influence condition) were randomly selected for each participant. The order of trials was pseudorandomized within each participant, such that there were never more than five consecutive trials from a single social-influence condition. In the control condition, instead of being shown the ratings of other people, participants were shown their first ratings and were then asked to rate the situations again. The purpose of the control condition was to check that there was no systematic difference between the age groups in terms of remembering their first rating and to find out the degree to which the participants in different age groups shifted their answers under no social influence. The timings of the stimulus presentation were identical for each trial in all three of the social-influence conditions.

### Statistical analysis

#### Risk-perception analysis

Differences in the first risk ratings across age groups were analyzed using a one-way analysis of variance (ANOVA) with post hoc pairwise comparisons (Bonferroni-adjusted alpha levels).

#### Social-influence analysis

We used linear mixed-effects models to investigate the degree to which participants changed their risk ratings in the direction of other people’s ratings (social-influence hypothesis) and whether the extent of this change depended on whether the social-influence group consisted of adults or teenagers (peer-influence hypothesis). These models incorporated: (a) fixed effects that reflected average effects within and differences between the three experimental conditions and (b) random effects that took into account individual variability in the effect of participants’ first rating on their second rating and individual variability in susceptibility to social influence.

The linear mixed-effects model was used to assess the dependence of a participant’s second rating on two main predictors: (a) the first rating and (b) the difference between the rating provided by the social-influence group and the participant’s first rating (Δrating). The latter predictor was used to assess the degree to which participants were influenced by the rating provided by the social-influence group. We were particularly interested in whether the effect of the provided rating was dependent on its source (i.e., teenager or adult social-influence group) and the participants’ ages. Therefore, the model also included interactions between Δrating and age, Δrating and social-influence group, and Δrating, age, and social-influence group.

Fixed effects were included for all the main and interaction factors in the model. In addition, the model included subject-specific random slopes for the first rating and Δrating. Effect coding was used for the social-influence groups (1 = adults; −1 = teenagers), whereas dummy coding was used for age group (with young adults as the baseline group). The model did not include an intercept, because an intercept not identical to 0 would mean that participants’ second rating always increased (or decreased). For the same reason, no main effects of social-influence group or age group were included. Preliminary analyses indicated that these effects were indeed redundant. For a more detailed description of the linear mixed-effects model analysis, see the Supplemental Material.

## Results

### Risk perception

**Fig. 2.**
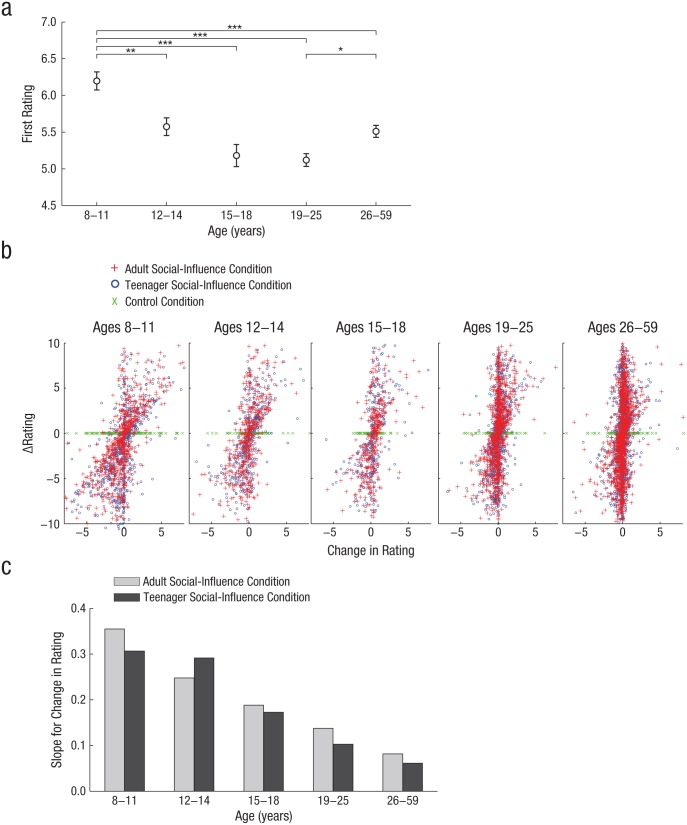
Results. The graph in (a) shows mean first risk rating as a function of age group: children (ages 8–11), young adolescents (ages 12–14), midadolescents (ages 15–18), young adults (ages 19–25), and adults (ages 26–59). The possible range of rating was from 0 (*low risk*) to 10 (*high risk*). Error bars represent ±1 *SE*. Asterisks indicate statistically significant differences between groups (*p < .05, **p < .01, ***p < .001; Bonferroni corrected). The scatter plots (b) illustrate the relationship between Δrating (the difference between the provided rating and the first rating) and change in rating (the difference between the second rating and the first rating) separately for each of the five age groups. Each symbol represents a single trial. Note that Δrating for the control condition was always zero. The graph in (c) presents the slopes for the average change in risk rating predicted by the difference between the provided rating and the first rating (Δrating). Results are shown separately for the adult social-influence condition and the teenager social-influence condition, for each age group. The slopes were calculated using estimates of the linear mixed-effect model analysis.

A one-way ANOVA was used to test whether the first risk rating differed across age groups. There was a main effect of age group on risk rating, *F*(4, 558) = 15.4, *p* < .001. Children rated the situations as more risky (*M* = 6.20, *SD* = 1.26) than did every other age group—children compared with young adolescents (*M* = 5.57, *SD* = 0.99): *t*(173) = 3.47, *p* < .01; children compared with midadolescents (*M* = 5.18, *SD* = 1.08): *t*(157) = 5.00, *p* < .001; children compared with young adults (*M* = 5.12, *SD* = 1.02): *t*(241) = 7.39, *p* < .001; and children compared with adults (*M* = 5.51, *SD* = 1.15): *t*(305) = 4.82, *p* < .001. In addition, adults rated the situations as more risky than did young adults, *t*(334) = −3.20, *p* < .05 (see Fig. 2a). No other pairwise comparison was significant—young adolescents compared with midadolescents: *t*(118) = 2.06, *p* = .58; young adolescents compared with young adults: *t*(202) = 3.03, *p* = .07; young adolescents compared with adults: *t*(266) = 0.40, *p* = 1.0; midadolescents compared with young adults: *t*(186) = 0.363, *p* = 1.0; and midadolescents compared with adults: *t*(250) = −1.86, *p* = .59.

Figure 2b shows scatter plots of raw data depicting the change in rating for all conditions and all age groups. The figure shows the decrease in social influence with age and illustrates that Δrating in the control condition was necessarily zero because the provided rating was simply the first rating. The linear mixed-effects model was used to estimate the second rating as a function of (a) the first rating, (b) Δrating, and (c) the interactions involving age group and social-influence group. Full results of the main linear mixed-effects analysis are reported in Tables 1 and 2. Omnibus *F* tests are comparable with those in traditional ANOVAs and show significance of all the components of the main linear mixed-effects model.

**Table 1.** Main Linear Mixed-Effects Model Predicting Change in Rating: Omnibus-Test Results

Predictor	*F* test	*p*
First rating	*F*(1, 666.32) = 32,129.69	< .001
ΔRating	*F*(1, 604.61) = 607.97	< .001
ΔRating × Age	*F*(4, 548.04) = 53.69	< .001
ΔRating × Social Influence	*F*(1, 9637.45) = 4.52	.034
ΔRating × Age × Social Influence	*F*(4, 9643.51) = 4.17	.002

**Table 2.** Main Linear Mixed-Effects Model Predicting Change in Rating: Fixed Effects

Fixed effect	*b*	*SE*	*t* test	*p*
First rating	0.98	0.01	*t*(665.48) = 179.25	< .001
ΔRating	0.12	0.01	*t*(565.93) = 8.53	< .001
ΔRating × Children	0.21	0.02	*t*(544.55) = 9.99	< .001
ΔRating × Young Adolescents	0.15	0.02	*t*(559.85) = 6.14	< .001
ΔRating × Midadolescents	0.06	0.03	*t*(541.26) = 2.28	.023
ΔRating × Adults	−0.05	0.02	*t*(545.82) = −2.69	.007
ΔRating × Social Influence	0.02	0.01	*t*(9619.13) = 2.70	.007
ΔRating × Children × Social Influence	0.01	0.01	*t*(9653.10) = 0.71	.478
ΔRating × Young Adolescents × Social Influence	−0.04	0.01	*t*(9633.64) = −3.44	.001
ΔRating × Midadolescents × Social Influence	−0.01	0.01	*t*(9609.38) = −0.78	.434
ΔRating × Adults × Social Influence	−0.01	0.01	*t*(9641.82) = −0.85	.393

Note: Effect coding was used for social-influence group (1 = adults; −1 = teenagers), whereas dummy coding was used for age group; young adults was used as the baseline group. The estimated standard deviations of the random effects were 0.14 (Δrating) and 0.08 (first rating). The standard deviation of the residuals was 1.02.

### Social-influence hypothesis

We used linear mixed-effects models to investigate the extent to which participants changed their risk ratings in the direction of others’ ratings. The extent of such changes did differ between age groups, as revealed by a significant two-way interaction between age group and Δrating, *F*(4, 548.04) = 53.69, *p* < .001. The slopes of the individual effects revealed that the slope for the first rating was close to 1, such that the other effects could clearly be interpreted as reflecting their influence on the change in rating. The slope of Δrating reflects its effect in the young adult (i.e., baseline) group and indicates that, on average, young adults (ages 19–25) changed their ratings in accordance with the ratings from the social-influence group, *t*(565.93) = 8.53, *p* < .001; the magnitude of this change was equal to about 12% of the difference between that rating and their first rating. This magnitude of the change for young adults differed from that in the other age groups: 33% for children (ages 8–11), 27% for young adolescents (ages 12–14), 18% for midadolescents (ages 15–18), and 7% for adults (ages 26–59).

In follow-up models, we repeated the analysis using each of the other age groups as the baseline group to assess whether the social-influence effect was significant for all age groups. These analyses showed that the effect of social influence was significant in all age groups—children: *t*(554.46) = 20.82, *p* < .001; young adolescents: *t*(573.69) = 13.47, *p* < .001; midadolescents: *t*(545.64) = 8.00, *p* < .001; young adults: *t*(565.93) = 8.53, *p* < .001; and adults: *t*(568.48) = 6.16, *p* < .001. Thus, all age groups changed their second ratings in the direction of the ratings provided by the social-influence group. This finding supports the social-influence hypothesis. However, the degree of social influence in young adults was different from that in every other age group: Children, *t*(544.55) = 9.99, *p* < .001; young adolescents, *t*(559.85) = 6.14, *p* < .001; and midadolescents, *t*(541.26) = 2.28, *p* < .05, were more strongly influenced by the provided ratings than were young adults, whereas older adults, *t*(545.82) = −2.69, *p* < .01, were less strongly influenced than were young adults (Fig. 2c).

### Peer-influence hypothesis

We investigated whether the extent of the change in risk ratings in the direction of others’ ratings depended on whether the social-influence group consisted of adults or teenagers. We found a significant three-way interaction of social-influence group, age group, and Δrating, *F*(4, 9643.51) = 4.17, *p* < .002, which indicates that social influence depended on social-influence group (teenagers or adults) and that this effect differed among the age groups. The effect of social-influence group was significant in young adults, who changed their ratings more after seeing the ratings from the adult social-influence group than after seeing ratings from the teenager social-influence group, *t*(9619.13) = 2.70, *p* < .01. The effect of social-influence group in young adults did not differ significantly from the effect of social-influence group in children, midadolescents, or adults. In contrast, the effect of social-influence group was significantly different in young adolescents, *t*(9633.64) = −3.44, *p* < .001 (Fig. 2c). Follow-up models for the peer-influence hypothesis showed that children and adults, like young adults, changed their ratings to a significantly greater extent when the social-influence group was adults rather than teenagers—children: *t*(9678.63) = 3.36, *p* < .001; adults: *t*(9605.25) = 1.951, *p* = .051. The effect in midadolescents was not significant, *t*(9605.25) = 0.77, *p* > .44, which indicates that midadolescents did not differentiate between the sources of social influence. Young adolescents, in contrast to all other age groups, were significantly more influenced by the teenager social-influence group than by the adult social-influence group, *t*(9640.28) = −2.33, *p* < .05.

## Discussion

In the present study, we demonstrated that risk perception was influenced by the risk ratings of other people and this social-influence effect decreased with age (social-influence hypothesis). Furthermore, this socially driven change in risk perception was dependent on the age of the participant and on whether the social-influence group consisted of teenagers or adults (peer-influence hypothesis). Specifically, although most age groups changed their ratings more toward those of the adult social-influence group than toward those of the teenager social-influence group, young adolescents showed the reverse effect, being more influenced by the teenager social-influence group than by the adult social-influence group.

The analysis of the first rating indicated that children (ages 8–11) rated the situations as significantly more risky than did adolescents and adults. The risk-ratings-by-age function followed a roughly U-shaped pattern (see Fig. 2a): There was a decrease in risk ratings between late childhood and midadolescence, followed by an increase between young adulthood and adulthood. Children might rate situations as more risky than other age groups for a number of reasons, including having less experience of risky situations (Halpern-Felsher et al., 2001) or being at greater risk in risky situations because of their physical or cognitive disadvantage (Anderson, 2002) relative to adolescents or adults. A similar decline in risk perception from late childhood to adulthood has been reported previously (Millstein & Halpern-Felsher, 2002), although a recent study found that young adults had the greatest likelihood of perceiving risky activities as a “good idea” (Shulman & Cauffman, 2014). Thus, this analysis fails to support the notion that adolescents (relative to other age groups) feel invulnerable to harm; this finding is in line with results from previous studies that found no evidence that adolescents are more likely than adults to underestimate or overestimate risk (Millstein & Halpern-Felsher, 2002).

A large body of literature has demonstrated that other people’s behavior can have a significant impact on one’s own behavior (Berns, Capra, Moore, & Noussair, 2010; Zaki et al., 2011). People change their behavior to fit in with other people. This social conformity effect has been studied extensively, and some research has suggested that children and adolescents show a higher susceptibility to social influence than adults do (Costanzo & Shaw, 1966; Hoving, Hamm, & Galvin, 1969). However, previous findings of age effects on social conformity are inconsistent (Costanzo & Shaw, 1966; Engelmann et al., 2012; Hoving et al., 1969; Iscoe, Williams, & Harvey, 1963; Walker & Andrade, 1996). A decrease in conformity from childhood to adolescence was found in several studies using monetary games (Engelmann et al., 2012) and perceptual judgment tasks (Hoving et al., 1969; Walker & Andrade, 1996), whereas in other studies using similar perceptual judgment tasks (Costanzo & Shaw, 1966; Hoving et al., 1969; Iscoe et al., 1963) and decision making in hypothetical scenarios (Berndt, 1979), the researchers reported an increase, with a peak in conformity in early adolescence to midadolescence. It has been suggested that ambiguous stimuli evoke stronger conformity effects, whereas with unambiguous stimuli, conformity declines with age (Walker & Andrade, 1996).

In the current study, participants were told that each trial had no correct or incorrect response. We found that all age groups showed a significant social-influence effect: All groups changed their risk ratings in the direction of the provided ratings. Children (age 8–11) exhibited the highest level of social conformity, indicated by a larger change in their risk ratings after being presented with other people’s ratings (Fig. 2c), and the social-influence effect decreased steadily between late childhood and adulthood. It is not possible to determine the underlying reason for the conformity we observed in our study. The provided ratings came from a group of people whom the participants had never met and never anticipated meeting; thus, there were no social consequences of conforming or not. It is possible that the social-influence effect found here is associated with a true reevaluation of risk; that is, participants may have taken into account the ratings of the social-influence group and changed their risk perception accordingly (informational conformity) rather than pursuing acceptance or simply being aware of social norms (normative conformity; Deutsch & Gerard, 1955). The decrease in conformity across age might be because older participants have more confidence in their own perception of a situation, perhaps because they have experienced the situation more often than children or adolescents have. Thus, our findings suggest that social influence on risk perception depends on the age of the participant, and we speculate that children and adolescents are more susceptible to informational influence from other people than adults are.

Our second hypothesis addressed the source of the social influence. Our findings suggest that social influence depends on age and is also critically dependent on the source of influence: Most age groups were more influenced by the adult social-influence group than by the teenager social-influence group, but the teenager social-influence group had a stronger impact on young adolescents. Thus, children, young adults, and adults changed their ratings to a greater degree toward the ratings from the adult social-influence group than toward the ratings from the teenager social-influence group. One explanation for this finding is that people in most age groups consider adults to be more competent and experienced role models than teenagers are. Previous studies have shown that expertise and status are strong predictors of social influence (Driskell & Mullen, 1990; Engelmann et al., 2012; Jetten et al., 2006). In contrast, young adolescents were more influenced by the teenager social-influence group than by the adult social-influence group. There was no significant difference between the adult and teenager social-influence groups regarding influence of midadolescents’ ratings.

An early study investigating decision making in social situations, in which parents and peers offered conflicting advice, found that although conformity to the advice of parents decreases with age, conformity to peers increases during adolescence (ages 12–17; Utech & Hoving, 1969). On the other hand, parental opinions continue to be important during adolescence: Adolescents rely on parental advice particularly when making decisions relevant for their futures (Commendador, 2010; Galotti & Mark, 1994). Our finding that the influence of the teenager social-influence group was stronger than the influence of the adult social-influence group in young adolescents might be due to additional normative influence in young adolescence. We speculate that adolescents seek to conform to the people of the same age, not because they trust the ratings of teenagers more than they trust the ratings of adults, but because they want to be accepted by their peer group (in this case, the teenager social-influence group). However, this is speculative, and future studies should attempt to further distinguish between normative and informational influence in adolescence. Young adolescents do not perceive situations as less risky than do other age groups but do tend to change their risk perception in the direction of the opinions of peers of a similar age; together, these two findings suggest that the target of public-health interventions should be adolescent social norms rather than (or in addition to) the potential health risks associated with certain situations and choices.

## Conclusion

In this study, we investigated social influence on risk perception across development from late childhood to adulthood. In general, all age groups changed their ratings to be in line with other people’s ratings, but there was a steady decline in this social conformity from late childhood through adulthood. This first finding might be explained by informational conformity: All groups trust in the ratings of the social-influence groups, but this trust is highest in children and decreases with age. Our second finding provided insights into the impact of different social-influence groups on risk perception in different age groups. Our results support the notion that early adolescence is a pivotal phase in which individuals begin to challenge the authority and experience of adults and place higher value on the opinions of other teenagers than on opinions of adults. The explanation for this trend might be heightened normative conformity in young adolescents, who place particular importance on being accepted by their peer group.

## Supplementary Material

Supplementary material
