# Supplementary material for: Social Influence on Risk Perception During Adolescence
Source: Psychol Sci. 2015 May;26(5):583–92. doi: 10.1177/0956797615569578 (PMC4426139; doi:10.1177/0956797615569578)
Supplement: Supplementary material [file DS_10.11770956797615569578.pdf]

DOI: 10.1177/0956797615569578

Supplemental Material

Experimental set-up

The experiment was performed in the Live Science research space at the London Science Museum. This is a 40m^2 circular self-contained space within the Who Am I Gallery. The research space contains a large clover-shaped desk with three ‘leaves’. On each ‘leaf’ is a computer. There were a maximum of three participants taking part at any one time, and there were always at least two experimenters present. Participants were not completely isolated but each participant was on their own when they took part in the task. Importantly, children were no more observed than any age group because parents were asked to sit on the bench around the outside of the research space, or take part in the experiment themselves, and were asked not to stand next to their child during the experiment.

Stimuli presentation

We generated a total of 79 risky situations from which 18 were randomly selected for each participant. An analysis of frequency revealed no difference in how many times a scenario was presented to any of the age groups (χ^2(312, N = 395) = 239.17, p = .99).

The provided ratings of the social influence group were presented at a random position on the risk rating scale (see Figure 1). However, the experiment was designed such that the provided ratings would be believable. First, we selected risky scenarios on the basis that they would generally be considered as moderately risky (neither very low nor very high risk) and, second, they would be associated with a variation in how risky they are perceived by different individuals. Thus, if the participant had rated a situation as low risk, for example, a provided rating that was high risk would be believable.

Linear mixed-effects model analysis

We used linear mixed-effects models to investigate how much participants change their risk

rating in the direction of others' ratings (social influence hypothesis), and whether the extent of this change depends on whether the social influence group is adults or teenagers (peer influence hypothesis). These models incorporate: (1) fixed effects that reflect average effects within and differences between the three experimental conditions and (2) random effects that take into account individual variability in the effect of participants' first rating on their second rating, and individual variability in susceptibility to social influence.

All models were estimated in R (R Core Team, 2004), using the lme4 (Bates, Maechler, & Bolker, 2013) and lmerTest (Kuznetsova, Brockhoff, & Christensen, 2013) packages. First, a sequential nested model comparison approach using Chi-square likelihood ratio tests was used to guide the selection of the best fitting model. Second, estimates of particular effects of interest in the global model were evaluated. Satterthwaite approximation was used to estimate the degrees of freedom. Supplementary data analysis was conducted in SPSS IBM version 21.

For data analysis, the *visual analogue scale* used to record participants' ratings was transformed to a 0.00 (low risk) to 10.00 (high risk) point scale. Variables were centred by subtracting the mean rating, which is standard practice in mixed effects modelling. Custom contrasts for hypothesis testing were used. In line with research on risk judgment (Shulman & Cauffman, 2013), the young adult age group was used as baseline in the main linear mixed-effects model.

The linear mixed-effects model was used to assess the dependence of the second rating (r2) made by the participant on two main predictors: (i) the first rating (r1) and (ii) the difference between the social influence group provided rating and the participant's first rating (Δrating). Δrating was used to assess the degree to which participants were influenced by the provided rating of the social influence group. Of particular interest was whether the effect of the provided risk perception depends on the source of the provided rating (social influence group

DOI: 10.1177/0956797615569578

teenagers or adults) and participants' age. Therefore, the model also included interactions between Δrating and age, Δrating and social influence group, and Δrating, age, and social influence group. The final model was based on 10,134 observations and 563 subjects, and had the following components:

r2 = r1 + Δrating + (Δrating x age group) + (Δrating x social influence group) + (Δrating x social influence group x age group)

Note that, because the first rating is included as a predictor, the model is similar to a model that predicts the difference between the second and first rating from the difference between the provided and first rating (subtracting r1 from both sides of the model equation). However, by estimating a slope for r1, the model is more general than a model with difference scores as dependent variables (which corresponds to fixing the slope of r1 to 1). The model did not include an intercept, as an intercept not identical to 0 would mean participants always increase (or decrease) their second rating. For the same reason, no main effects of social influence group or age group were included. Preliminary analyses indicated that these effects were indeed redundant.

Fixed effects were included for all the (main and interaction) factors in the model. In addition, the model included subject-specific random slopes for the difference between provided rating and first rating (Δrating) and the first rating. Effect coding was used for social influence group (1 = adults; -1 = teenagers), while dummy coding was used for age group (with young adults as the baseline group). In the control condition, Δrating is necessarily zero because the provided rating is simply a re-presentation of the first rating. Therefore, our linear mixed-effects model is effectively reduced to a simple model in the control condition, where the second rating depends only on the first rating. Thus data from the control condition will reflect the extent to which second and first ratings differ irrespective of the provided rating. Although data from the control condition will not affect estimates of the social

influence effects, it will inform both the fixed and random effects of the first rating, as well as the estimates of the error variance (which reflects differences between the first and second rating not explained by social influence). The purpose of the control condition was to check that there was no systematic difference between the age groups in terms of remembering their first rating, and to find out how much the age groups shifted their answers under no social influence. Using a mixed model repeated measures ANOVA to investigate differences in the control condition, we found no significant main effect of rating (first rating, second rating) ($F(1, 558) = .827$, $p = .36$) and no interaction between rating and age group (5 levels) ($F(4, 558) = .567$, $p = .69$). This suggests that there was no significant change in rating under no social influence (the control condition). Figure 2B illustrates higher variance of children's responses in the control condition. However, our findings cannot be explained by differences in variance between the groups as additional analyses showed that accounting for heteroscedasticity in the error variance in the fixed effects model and including the difference in control variance as an additional predictor in the model, did not change the key statistical results (See Table S1): The two-way interaction between age group and Δrating ($F(4,9560) = 43.19$, $p < .001$), as well as the three-way interaction between social influence group, age group and Δrating ($F(4,9560) = 3.25$, $p < .01$), remained significant in the additional analyses.

References SI

Bates, D. J., Maechler, M., & Bolker, B. (2013). lme4: Linear-mixed effects models using S4 classes. Retrieved from http://CRAN.R-project.org/package=lme4

Kuznetsova, A., Brockhoff, P. B., & Christensen, R. H. B. (2013). lmerTest: Tests for random and fixed effects for linear mixed effect models (lmer objects of lme4 package).

R Core Team. (2004). R: A language and environment for statistical computing. Retrieved from http://www.R-project.org

Shulman, E. P., & Cauffman, E. (2013). Deciding in the Dark: Age Differences in Intuitive Risk Judgment. *Developmental Psychology*. doi:10.1037/a0032778

DOI: 10.1177/0956797615569578

Table S1

Additional linear mixed-effects model. (A.) Omnibus test results. Table shows omnibus test with degrees of freedom (df), F values and p-values. (B.) **Slopes and test results**. Table shows fixed effects are summarized showing estimates, standard error (SE), df, t-values and p-values for the model fixed predictors and their interactions. The random effects summary table shows group, name of the variable, variance, and standard deviation (SD). Abbreviation: first rating (r1); Δrating (Δr); age group (age); csd (control variation as standard deviation), social influence group (social influence).

**A. Omnibus test**					
	**numerator df**	**denominator df**	**F**	**p**	
r1	1	9561	51176.31	<.001	***
Δr	1	9561	107.54	<.001	***
Δr:age	4	9561	43.19	<.001	***
Δr:social influence	1	9561	10.16	.001	**
Δr:age:social influence	4	9561	3.24	.01	**

**B. Slopes and tests of the individual fixed effects**						
**Fixed effects**	**Estimate**	**SE**	**df**	**t**	**p**	
r1	0.99	0.01	9561	226.22	<.001	***
Δr	0.13	0.01	9561	10.37	<.001	***
Δr:csd	0.04	0.01	9561	5.99	<.001	***
Δr:children	0.19	0.02	9561	9.08	<.001	***
Δr:young adol	0.13	0.02	9561	5.99	<.001	***
Δr:mid-adol	0.06	0.02	9561	2.61	.01	**
Δr:adults	-0.04	0.02	9561	-2.73	.006	**
Δr:social influence	0.02	0.01	9561	3.19	.001	**
Δr:children:social influence	0.01	0.01	9561	0.76	.448	
Δr:young adol:social influence	-0.04	0.01	9561	-3.26	.001	**
Δr:mid-adol:social influence	-0.01	0.01	9561	-0.76	.447	
Δr:adults:social influence	-0.01	0.01	9561	-1.03	.304	

**Random effects**	**Variance**	**SD**
Δr	0.015	0.121
r1	0.002	0.045
residual	0.657	0.811

DOI: 10.1177/0956797615569578

List of Stimuli

Playing with matches
Riding a bike without a helmet
Skateboarding without protection
Riding a bike hands-free
Climbing on a big tree
Swinging as high as possible
Skydiving for the first time
Crossing a railway track
Jumping off a roof
Swimming alone in a lake
Walking alone at night
Scuba diving for the first time
Drinking more than three alcoholic drinks
Riding a bike with two people
Driving without a seatbelt
Texting while biking
Calling while biking
Crossing a street on a red light
Walking through a dark alley
Sticking a finger into a plug socket
Bungee jumping
Riding a bike without brakes
Parachute jumping
Performing chemical experiments without goggles
Climbing a flimsy ladder
Swimming during a thunderstorm
Diving off a 10 meter platform
Skiing without a helmet
Snowboarding without protection
Climbing on scaffolding
Getting a tattoo
Getting piercings
Stroking a stray dog
Playing with fire
Picking up broken glass with bare hands
Standing under a tree during a thunderstorm
Eating self-picked mushrooms
Playing with fireworks
Cycling without lights in the dark
Swatting a wasp
Driving very fast
Picking up a dead pigeon
Sunbathing without sun block
Eating rotten food
Drinking tap water in a foreign country
Running down the stairs
Drinking hot beverages
Listening to loud music
Trespassing on private property
Leaving cooking unattended
Not locking the front door
Eating unwashed fruits
Eating raw eggs
Swimming in a strong current
Crossing the road while texting
Not wearing a raincoat in a storm
Going out in the cold with wet hair
Replacing a light bulb without turning off the fuse
Not disinfecting a wound
Eating wild berries
Chopping wood with an axe
Running with untied shoe laces
Stealing honey out of a bee hive
Ice-skating on a half-frozen lake
Sledging in the dark
Rock climbing without ropes
Feeding a snarling dog
Riding with a drunk driver
Balancing on a high wall
Hitch-hiking
Playing contact sports
Taking pain relievers
Running with scissors
Staring directly into the sun
Running on a slippery surface
Not wearing gloves when it is cold outside
Using an elevator during a fire alarm
Playing rugby
Getting into a fist fight
